# Efficacy and survival of nivolumab treatment for recurrent/unresectable esophageal squamous-cell carcinoma: real-world clinical data from a large multi-institutional cohort

**DOI:** 10.1007/s10388-024-01056-w

**Published:** 2024-05-08

**Authors:** Tomoki Makino, Shigeto Nakai, Kota Momose, Kotaro Yamashita, Koji Tanaka, Hiroshi Miyata, Sachiko Yamamoto, Masaaki Motoori, Yutaka Kimura, Yuki Ushimaru, Motohiro Hirao, Jin Matsuyama, Yusuke Akamaru, Yukinori Kurokawa, Hidetoshi Eguchi, Yuichiro Doki

**Affiliations:** 1 Department of Gastroenterological Surgery, Graduate School of Medicine, Osaka University, 2-2-E2, Yamada-Oka, Suita, Osaka 565-0871 Japan; 2 Department of Gastroenterological Surgery, Osaka International Cancer Institute, Osaka, Japan; 3 Department of Gastrointestinal Oncology, Osaka International Cancer Institute, Osaka, Osaka Japan; 4 Department of Surgery, Osaka General Medical Center, Osaka, Japan; 5 Department of Gastroenterological Surgery, Kindai University Nara Hospital, Nara, Japan; 6 Department of Surgery, Sakai City Medical Center, Osaka, Japan; 7 Department of Surgery, National Hospital Organization Osaka National Hospital, Osaka, Japan; 8 Department of Gastroenterological Surgery, Higashiosaka City Medical Center, Osaka, Japan; 9 Department of Surgery, Osaka Rosai Hospital, Osaka, Japan

**Keywords:** Nivolumab, Esophageal cancer, Survival, Response rate

## Abstract

**Background:**

Real-world clinical outcomes of and prognostic factors for nivolumab treatment for esophageal squamous-cell carcinoma (ESCC) remain unclear. This study aimed to evaluate real-world outcomes of nivolumab monotherapy in association with relevant clinical parameters in recurrent/unresectable advanced ESCC patients.

**Methods:**

This population-based multicenter cohort study included a total of 282 patients from 15 institutions with recurrent/unresectable advanced ESCC who received nivolumab as a second-line or later therapy between 2014 and 2022. Data, including the best overall response, progression-free survival (PFS), and overall survival (OS), were retrospectively collected from these patients.

**Results:**

Objective response and disease control rates were 17.0% and 47.9%, respectively. The clinical response to nivolumab treatment significantly correlated with development of overall immune-related adverse events (*P* < .0001), including rash (*P* < .0001), hypothyroidism (*P* = .03), and interstitial pneumonia (*P* = .004). Organ-specific best response rates were 20.6% in lymph nodes, 17.4% in lungs, 15.4% in pleural dissemination, and 13.6% in primary lesions. In terms of patient survival, the median OS and PFS was 10.9 and 2.4 months, respectively. Univariate analysis of OS revealed that performance status (PS; *P* < .0001), number of metastatic organs (*P* = .019), C-reactive protein-to-albumin ratio (CAR; *P* < .0001), neutrophil–lymphocyte ratio (*P* = .001), and PMI (*P* = .024) were significant. Multivariate analysis further identified CAR [hazard ratio (HR) = 1.61, 95% confidence interval (CI) 1.15–2.25, *P* = .0053)] in addition to PS (HR = 1.65, 95% CI 1.23–2.22, *P* = .0008) as independent prognostic parameters.

**Conclusions:**

CAR and PS before nivolumab treatment are useful in predicting long-term survival in recurrent/unresectable advanced ESCC patients with second-line or later nivolumab treatment.

**Trial Registration:**

UMIN000040462

**Supplementary Information:**

The online version contains supplementary material available at 10.1007/s10388-024-01056-w.

## Introduction

Esophageal cancer is the seventh leading cause of cancer mortality worldwide. Esophageal squamous-cell carcinoma (ESCC) is the most common histological subtype of esophageal cancer, accounting for approximately 90% of all cases worldwide [1, 2]. Many ESCCs are unresectable at diagnosis, and over half of patients treated with curative intent eventually have a relapse [3–11]. Patients with unresectable or metastatic ESCC are known to have a poor prognosis, with a median overall survival (OS) of 8–10 months. Therefore, the development of novel therapeutic agents is urgently required [12].

Inhibitors of immune-checkpoint protein PD-1 enhance the antitumor activity of T cells by blocking the interaction between the PD-1 receptor and its ligands [13, 14]. The efficacy and safety of human monoclonal anti-PD-1 antibody nivolumab for the treatment of unresectable advanced or recurrent ESCC was demonstrated in the ATTRACTION-1 trial of patients with advanced ESCC refractory or intolerant to fluoropyrimidine-based, platinum-based, and taxane-based chemotherapy [15]. The superiority of nivolumab over taxane was then demonstrated in the ATTRACTION-3 trial [16]. Accordingly, nivolumab has been approved as a new second-line treatment for patients with advanced ESCC resistant to fluoropyrimidine and platinum drugs. However, in these trials, approximately 50% of patients treated with PD-1 monoclonal antibody for ESCC exhibited progressive disease [15, 16]. As such, the identification of predictive biomarkers to select patients who will benefit from PD-1 blockade is urgently needed [17].

In clinical practice, anti-PD-1 antibody may also be administered in patients who do not meet the eligibility criteria for clinical trials, including patients with poor performance status (PS) or severe comorbidities or elderly populations. Although drug efficacy needs to be assessed in both clinical trials and real-world settings, real-world data on the efficacy and survival of nivolumab monotherapy for unresectable advanced or recurrent ESCC in clinical practice are very limited [18]. In addition, since the CheckMate 648 trials demonstrated the efficacy of nivolumab as a first-line therapy [19], combination chemotherapy with nivolumab or a dual immune checkpoint inhibitors is increasingly being used in clinical settings; therefore, the availability of prospective data for nivolumab monotherapy is limited. To the best of our knowledge, the current study is the largest set of real-world data on safety and outcomes in unresectable/recurrent ESCC patients treated with nivolumab as a second-line or later monotherapy.

## Patients and methods

### Patients

This cohort study included patients with unresectable or recurrent ESCC who had been treated or were scheduled to be treated with nivolumab as second-line or later therapy between 2014 and 2022 at any of the 15 institutions of the clinical study group of osaka university, upper gastrointestinal surgery group. The eligibility criteria were age ≥ 20 years and histologically diagnosed squamous-cell carcinoma of the esophagus refractory or intolerant to one or more previous chemotherapy regimens. Patients who were previously treated with any immune-checkpoint inhibitor other than nivolumab were ineligible. Patients who had synchronous or metachronous (within 5 years) malignancy other than carcinoma in situ or mucosal carcinoma at the start of nivolumab treatment were excluded. Patients provided written informed consent before enrollment. Only for patients who were dead or lost to follow up was informed consent not required. The study was approved by the institutional review boards of all participating institutions. This study is registered with UMIN Clinical Trials Registry under number UMIN000040462.

### Evaluation of tumor response and adverse events

Although a follow-up schedule was not specified in this study, the efficacy evaluation was conducted every 6–8 weeks in most of the patients. Tumor response was assessed according to Response Evaluation Criteria in Solid Tumors version 1.1 (RECIST v1.1). A minimum interval of 6 weeks between two measurements was required for determination of a complete response (CR), partial response (PR), or stable disease (SD) [20, 21]. Non-evaluable (NE) patients were regarded as non-responders. The response rate was assessed only in patients with measurable lesions and was defined as the proportion of patients with a best overall response of a CR or PR; both groups were considered to be responders. Adverse events (AEs) were assessed throughout the treatment and follow-up periods according to the national cancer institute common terminology criteria for adverse events (CTCAE) version 4.0. In this study, immune-related adverse events (irAE) were defined as a set of side effects (CTCAE > grade 2) in the patients receiving immune-checkpoint inhibitors similar to autoimmune responses [22].

### Psoas muscle index measured by computed tomography

Psoas muscle mass was measured on computed tomography (CT) scans, which were performed before nivolumab treatment. Briefly, both sides of the psoas muscle region were selected automatically and the cross-sectional psoas muscle area (cm^2^) measured at the level of the third lumbar vertebra (L3). The psoas muscle index (PMI) was calculated by adjusting for patient height as follows: PMI (cm^2^/m^2^) = total psoas area at L3 (cm^2^)/height^2^ (m^2^). The cut-off values for PMI were set at 6.36 cm^2^/m^2^ for males and 3.92 cm^2^/m^2^ for females [23, 24].

### Statistical analysis

The relationships between clinicopathological characteristics and tumor response status were analyzed using the Chi-squared test for categorical variables. Progression-free survival (PFS) was defined as the interval from the date of the first administration of nivolumab to the date of disease progression or death from any cause. OS was defined as the interval from the date of the first administration of nivolumab to the date of death due to any cause. Survival rates were estimated using the Kaplan–Meier method and compared by the log-rank test. The prognostic variables that were significantly associated with OS in the univariate analyses were further assessed in multivariate Cox proportional hazard model analyses. *P* < 0.05 was considered to indicate significance.

## Results

### Patient baseline characteristics

Baseline characteristics are provided in eTable 1. A total of 218 patients (77.3%) were male and the median age at immune-checkpoint inhibition initiation was 69 years (range 32–89 years). All enrolled patients had ESCC. Nearly half of the present cohort (52.5%) had an Eastern cooperative oncology group (ECOG) PS of 0. The number of unresectable advanced and recurrent cases was 130 (46.1%) and 152 (53.9%), respectively. The number of metastatic organs was 1 in 131 (46.4%) cases and 2 in 91 (32.3%) cases. All patients had received previous systemic anticancer therapy except 6 (2.1%) cases; 153 (54.3%) and 125 (44.3%) out of 282 patients had previous surgery and radiotherapy, respectively.

### Clinical response to nivolumab treatment

**Fig. 1:**
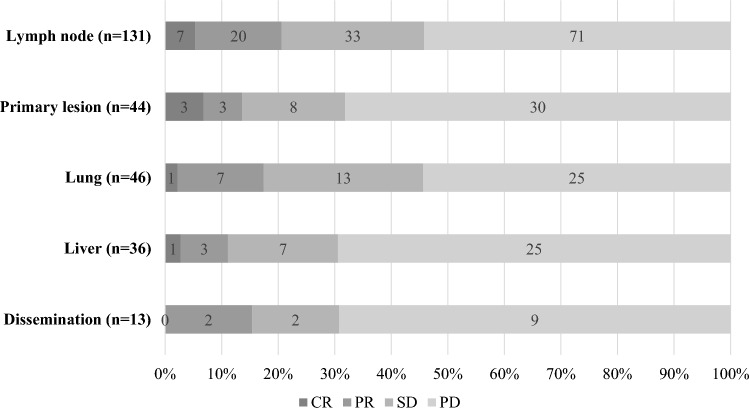
Organ-specific responses to nivolumab. *CR* complete response, *PR* partial response, *SD* stable disease, *PD* progressive disease

Among 282 patients with measurable lesions, the best overall response was CR in 8 (2.8%) patients, PR in 40 (14.2%) patients, SD in 87 (30.9%) patients, progressive disease (PD) in 140 (49.6%) patients, and non-evaluable (NE) in 7 (2.5%) patients. Thus, the objective response and disease control rates in this study were 17.0% (48/282) and 47.9% (135/282), respectively (Table 1). The median duration of response was 19.8 months [95% confidence interval (CI): 13.7–27.9]. With respect to organ-specific response evaluation, the best response rates (CR + PR) were 20.6% (27/131) in lymph nodes, 17.4% (8/46) in lungs, 15.4% (2/13) in pleural dissemination, and 13.6% (6/44) in primary lesions, whereas the highest PD rates were 69.4% (25/36) in liver, 69.2% (9/13) in pleural dissemination, 68.1% (30/44) in primary lesions, and 55.6% (5/9) in bone (Fig. 1). A Cox multivariate analysis using patient background parameters for predicting no response (SD and PD) to nivolumab revealed that the PS (*P* = 0.040), C-reactive protein-to-albumin ratio (CAR; *P* = 0.016), and neutrophil–lymphocyte ratio (*P* = 0.013) were significant. Remaekably, multivariate analysis identified PMI to be an independent predictors of nivolumab response in the multivariate analysis (HR = 2.00, 95% CI 1.02–3.93, *P* = 0.043; eTable 2).

**Table 1:** Antitumor activity

Outcome	*N*=282
Objective response	48 (17.0%)
Complete response	8 (2.8%)
Partial response	40 (14.2%)
Stable disease	87 (30.9%)
Progressive disease	140 (49.6%)
Not evaluable	7 (2.5%)
Disease control	135 (47.9%)
Duration of response, median (95% CI), months	19.8 (13.7–27.9)

*CI* confidence interval

### Immune-related adverse events and correlation with the response to nivolumab

Details of treatment-related AEs (> grade 2) are summarized in eTable 3. Common treatment-related AEs were rash (4.3%), hypothyroidism (4.3%), interstitial lung disease (3.5%), lung infection (3.2%), and diarrhea (1.4%). Compared with non-responders (SD + PD), nivolumab responders (CR + PR) more often developed irAEs (> grade 2; *P* < 0.0001), including rash (> grade 2; *P* < 0.0001), hypothyroidism (> grade 2; *P* = 0.017), and interstitial lung disease (> grade 2; *P* = 0.030; Table 2).

**Table 2:** Correlation between Response to Nivolumab and Immune-related Adverse Events

	Responders	Non-responders	*P* value
	(CR/PR, *n*=48)	(SD/PD, *n*=227)	
Immune-related adverse events, no. (%)			
All events	20 (41.7%)	28 (12.3%)	<.0001
Rash	8 (16.7%)	4 (1.8%)	<.0001
Hypothyroidism	4 (8.3%)	8 (3.5%)	0.017
Interstitial lung disease	4 (8.3%)	5 (2.2%)	0.03
Diarrhea	0	4 (1.8%)	0.21
Hepatic function abnormal	1 (2.1%)	3 (1.3%)	0.62
Hyponatremia	2 (4.2%)	1 (0.4%)	0.08
Glucose intolerance	1 (2.1%)	1 (0.4%)	0.29

*CR* complete response, *PR* partial response, *SD* stable disease, *PD* progressive disease

### Survival analysis

**Fig. 2:**
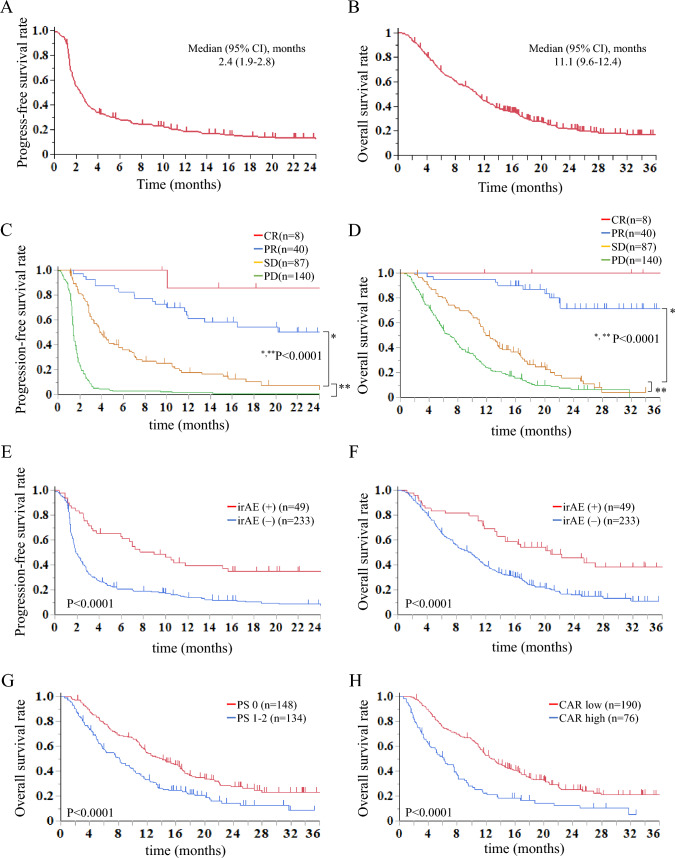
Kaplan–Meier analyses of survival for all patients (*n* = 282); **A** Overall survival. **B** Progression-free survival. Kaplan–Meier progression-free and overall survival curves for 275 patients who had measurable lesions; **C** Progression-free survival and **D** overall survival in complete response (CR, red) or partial response (PR, blue; *n* = 25), stable disease (SD, yellow; *n* = 18), progressive disease (PD, green). **E** Progression-free survival and (F) overall survival in patients with immune-related adverse events (irAE( +) = red) and without irAEs (irAE(−) = blue). Kaplan–Meier overall survival curves according to **G** performance status (0 = red, 1–2 = blue), and **H** C-reactive protein-to-albumin ratio (CAR; low = red, high = blue) (color figure online)

The median follow-up periods for PFS and OS in the censored patients were 16.2 months and 20.0 months, respectively. The median PFS and OS for nivolumab were 2.4 months (95% CI 1.9–2.8 months) and 11.1 months (95% CI 9.6–12.4 months), respectively (Fig. 2A-B). Kaplan–Meier survival curves for PFS and OS in the 275 patients according to clinical response are shown in Fig. 2C-D. The median PFS for nivolumab in the CR, PR, SD, and PD patients was 71.6, not reached, 4.0, and 1.4 months, respectively. There were significant differences in PFS between PR and SD (*P* < 0.0001), SD and PD (*P* < 0.0001; Fig. 2C). Nivolumab responders (CR + PR) had significantly longer PFS than non-responders (SD + PD) (1 year PFS rate: 65.4% and 7.6%, *P* < 0.0001; Fig. 2E). Furthermore, the median OS for nivolumab in CR, PR, SD, and PD patients was not reached, 40.8, 12.4, and 6.9 months, respectively. There were significant differences in OS between PR and SD (*P* < 0.0001) and SD and PD (*P* < 0.0001; Fig. 2D). Nivolumab responders (CR + PR) also has significantly longer OS than non-responders (SD + PD) (1 year OS rate: 93.7% and 34.1%, respectively; Fig. 2F). Patients who developed irAEs (> grade 2) had significantly better PFS and OS than those without irAEs (1 year PFS rate: 33.8% vs. 14.2%, *P* < 0.0001; 1 year OS rate: 62.8% vs. 39.3%; *P* = 0.0002; Fig. 2E, F). Among 282 patients, 265 (93.9%) patients received at least one subsequent treatment. The most common regimens were docetaxel (38.8%), paclitaxel (28.2%), and S-1 (17.2%).

A Cox univariate analysis for OS with clinicopathological covariables of patient background revealed that the PS, number of metastatic organs, CAR, neutrophil–lymphocyte ratio, and PMI were significant (Table 3, Fig. 2G, H). Multivariate analysis further identified PS and CAR as independent prognostic parameters of OS (Table 3).

**Table 3:** Univariate and multivariate analyses for overall survival

Variable	Category	Univariate analysis		Multivariate analysis	
		HR (95% CI)	*P* value	HR (95% CI)	*P* value
Age	≥ 70 years	0.90 (0.65–1.24)	.90		
Sex	Male	0.93 (0.64–1.34)	.70		
Performance status	1–3	1.74 (1.33–2.30)	< .0001	1.65 (1.23–2.22)	**.0008**
History of smoking	Yes	0.81 (0.57–1.16)	.26		
Previous surgery	No	1.22 (0.93–1.60)	.14		
Previous radiotherapy	Yes	0.98 (0.75–1.29)	.91		
Number of previous chemotherapy rounds	> 3	1.26 (0.82–1.91)	.28		
Number of metastatic organs	> 2	1.37 (1.05–1.80)	.019	1.32 (0.99–1.77)	.064
BMI (cutoff 18.5)	High	1.16 (0.83–1.69)	.37		
CAR (cutoff 0.5)	High	2.63 (1.89–3.65)	< .0001	1.61 (1.15–2.25)	**.0053**
NLR (cutoff 5)	High	1.71 (1.24–2.35)	.0010	1.32 (0.96–1.81)	.083
PMI	Low	1.38 (1.04–1.85)	.024	1.17 (0.86–1.59)	.31

Boldface *P* value <0.05

*BMI* body mass index, *CAR* C-reactive protein-to-albumin ratio, *NLR* neutrophil–lymphocyte ratio, *PNI* prognostic nutritional index, *PMI* psoas muscle index

## Discussion

In the present multi-institutional observation study with a large cohort, we demonstrated real-world outcomes of nivolumab treatment for ESCC. The objective response and disease control rates were 17.0% and 47.9%, respectively, and the lymph nodes and lungs had a relatively higher organ-specific response rate. The response to nivolumab significantly correlated with irAE development (> grade 2), including rash and interstitial lung disease. In terms of patient survival, the median PFS and OS were 2.4 and 10.9 months, respectively. Among various patient background parameters, CAR in addition to PS before nivolumab treatment were identified as independent prognostic parameters by multivariate analysis of OS. To the best of our knowledge, this study represents the largest real-world experience of second-line or later PD-1 antibody treatment for unresectable/recurrent ESCC.

In the present study, the median OS of all patients treated with nivolumab was 11.1 months, which is almost identical to that (10.9 months) of the ATTRACTION-3 study [16], whereas the PFS (2.4 months) of our cohort was slightly better. This is deemed a favorable result considering that, unlike the ATTRACTION-3 trial, this study included real-world data on patients with diverse profiles, such as those with poor PS, those with comorbidities, and those receiving multiple treatments. In addition, regarding AEs of nivolumab, no new safety signals for nivolumab were identified, and the safety profile presented in this study is consistent with or even better than the profile previously established in patients with ESCC and other solid tumors [15, 16]. In terms of the response to nivolumab, the present study showed a comparable objective response (17.0%) and disease control rate (47.9%) to that of ATTRACTION-3 [16]. According to evaluations of organ-specific responses, lymph nodes (20.6%) had the best objective response, followed by lungs (17.4%), whereas the liver had the highest PD rate (69.4%) among various organs. This trend was also supported by the previous reports focusing on the response at each metastatic site in different cancer types [25–28].

Recently, several studies have highlighted the utility of immune prognostic scores to appreciate the importance of routine laboratory parameters, because they are easily accessible and sensitive nutrition-based biomarkers [29, 30]. Although these biomarkers, including CRP, CAR, GPS, PNI, and NLR, have shown prognostic value in various treatment settings for diverse cancers, including esophageal cancer [30–34], they have gained more attention recently with regard to immune-oncology treatment. In fact, our study demonstrated that the baseline CAR, which reflects both the inflammatory and nutritional status, could be a potential predictor of OS in ESCC patients treated with PD-1 inhibitor. The present results were also supported by the previous report with a smaller sample size of advanced/recurrent ESCC treated with nivolumab [35]. CRP, which is elevated by pro-inflammatory cytokines, including IL-1, IL-8, and IL-6, has a profound suppressive effect on adaptive immunity by impacting both effector T cells and antigen presentation [36]. Moreover, CRP is associated with a poor clinical outcome for various cancers treated with immune-checkpoint inhibitors (ICIs), including melanoma and non–small cell lung cancer (NSCLC) [16]. Hypoalbuminemia has also been associated with impaired systemic cell-mediated immune responses, such as macrophage activation and granuloma formation, as well as poor prognosis in patients with cancer receiving various treatments [29, 30, 33]. Remarkably, we identified CAR as an important prognostic factor in cT4b ESCC patients who underwent curative resection at our institution [37]. Taken together, the evidence indicates that the CAR could precisely reflect immuno-nutrition status, which could be closely correlated with patient survival, particularly in advanced ESCC cases.

This study had several limitations. First, the present study used a retrospective design. However, it was a multicenter cohort study that included as many as 15 institutions, and the data from consecutive ESCC patients treated with nivolumab were obtained from every institution, minimizing selection bias. However, the patient follow-up schedule was not specified due to the retrospective nature, which may have affected PFS outcomes. Second, in the present study, we did not evaluate PD-L1 status (i.e., tumor proportion score or combined positive score) in association with the nivolumab response or patient survival. Although PD-L1 was suggested as a biomarker for pembrolizumab in the KEYNOTE-181 trial, patients with low or undetectable PD-L1 expression may still gain clinical benefit from pembrolizumab, whereas a considerable proportion of patients with high PD-L1 expression may not [38]. Therefore, the current study focused on clinical and routine laboratory parameters other than PD-L1 to identify predictive biomarkers for ICIs in ESCC. Using clinical samples from the present cohort, a biomarker study is currently underway with comprehensive molecular or pathological analyses of tumors in addition to host factors to establish tailor-made ICI treatments for ESCC [8, 39]. Third, there may be controversies regarding the cut-off values of NLR, CAR, and PNI. Nonetheless, we believe that this multicenter study with a large series provides important information that may ultimately lead to improved clinical outcomes in unresectable/recurrent ESCC.

In conclusion, the present multicenter observational study showed real-world outcomes of nivolumab as second-line or later treatment for unresectable advanced/recurrent ESCC. CAR and PS before nivolumab treatment are useful for predicting the long-term survival of ESCC patients.

### Supplementary Information

Below is the link to the electronic supplementary material.

Supplementary file1 (DOCX 17 kb)

Supplementary file2 (DOCX 18 kb)

Supplementary file3 (DOCX 15 kb)

## Data Availability

All data and materials are available in this study.
