# Supplementary material for: Efficacy and survival of nivolumab treatment for recurrent/unresectable esophageal squamous-cell carcinoma: real-world clinical data from a large multi-institutional cohort
Source: Esophagus. 2024 May 8;21(3):319–27. doi: 10.1007/s10388-024-01056-w (PMC11199269; doi:10.1007/s10388-024-01056-w)
Supplement: Supplementary file 1 — Supplementary file1 (DOCX 17 kb) [file 10388_2024_1056_MOESM1_ESM.docx]

eTable 1. Patient characteristics

| Characteristic | Value (n=282) |
| --- | --- |
| Age, median (range), years | 69（32-89) |
| Gender, no. (%)  　Male / Female | 218 (77.3) / 64 (22.7) |
| Performance status, no. (%)  　0 / 1 / 2 / 3 | 148 (52.5) / 117 (41.5) / 15 (5.3) / 2 (0.7) |
| History of smoking, no. (%)  　Yes / No | 197 (69.8) / 85 (30.2) |
| Unresectable/recurrent, no. (%) | 130 (46.1) / 152 (53.9) |
| Number of previous chemotherapy rounds, no. (%)  　0 / 1 / 2 / >3 | 6 (2.1) / 163 (57.8) / 73 (25.9) / 40 (14.2) |
| Number of organs with metastases, no. (%)  　0 / 1 / 2 / >3 | 15 (5.3) / 131 (46.4) / 91 (32.3) / 45 (16.0) |
| Previous surgery, no. (%)  　Yes / No | 153 (54.3) / 129 (45.7) |
| Previous radiotherapy, no. (%)  　Yes / No | 125 (44.3) / 157 (55.7) |

Table 1. Patient characteristics (continued)

| Characteristic | Value (n=282) |
| --- | --- |
| BMI, median (range), kg/m^2^ | 18.9 (13.3-35.4) |
| Serum albumin, median (range), g/dl | 3.7 (2.1-4.6) |
| GPS score^*^, no. (%)  　0 / 1 / 2 | 130 (48.7) / 79 (29.6) / 58 (21.7) |
| CAR, median (range) | 0.15 (0.02-11.57) |
| NLR, median (range) | 3.73 (0.32-95.5) |
| PNI, median (range) | 42 (24.3-70.7) |
| PMI, median (range), cm^2^/m^2^ | 5.23 (1.21-9.58) |

BMI; body mass index, GPS score 0; Alb ≥ 3.5 and CRP ≤ 1.0, 1; Alb < 3.5 or CRP＞1.0, 2; Alb＜3.5 and CRP＞1.0, CAR; C-reactive protein-to-albumin ratio, NLR; neutrophil–lymphocyte ratio, PNI; prognostic nutritional index, PMI; psoas muscle index,
