# Supplementary material for: Efficacy and survival of nivolumab treatment for recurrent/unresectable esophageal squamous-cell carcinoma: real-world clinical data from a large multi-institutional cohort
Source: Esophagus. 2024 May 8;21(3):319–27. doi: 10.1007/s10388-024-01056-w (PMC11199269; doi:10.1007/s10388-024-01056-w)
Supplement: Supplementary file 2 — Supplementary file2 (DOCX 18 kb) [file 10388_2024_1056_MOESM2_ESM.docx]

eTable 2. Univariate and Multivariate Analyses for no response to nivolumab

| Variable | Category | Univariate analysis | | Multivariate analysis | |
| --- | --- | --- | --- | --- | --- |
|  |  | HR (95% CI) | *P* value | HR (95% CI) | *P* value |
| Age | ≥70 years | 0.70 (0.34-1.43) | .33 |  |  |
| Sex | Male | 1.33 (0.61-3.03) | .44 |  |  |
| Performance status | 1-3 | 1.98 (1.03-3.81) | .040 | 1.45 (0.72-2.91) | .30 |
| History of smoking | Yes | 1.57 (0.50-2.45) | .17 |  |  |
| Previous surgery | No | 1.15 (0.62-2.16) | .65 |  |  |
| Previous radiotherapy | Yes | 1.03 (0.55-1.03) | .93 |  |  |
| Number of previous chemotherapy rounds | >3 | 1.71 (0.79-3.81) | .18 |  |  |
| Number of organs with metastases | >2 | 1.08 (0.58-2.02) | .80 |  |  |
| BMI (cut off 18.5) | High | 1.11 (0.53-2.31) | .76 |  |  |
| CAR (cut off 0.5) | High | 3.03 (1.22-7.97) | .016 | 1.85 (0.70-4.93) | .26 |
| NLR (cut off 5) | High | 2.75 (1.09-6.92) | .013 | 1.81 (0.76-4.32) | .17 |
| PMI | Low | 2.26 (1.20-4.26) | .011 | 2.00 (1.02-3.93) | **.043** |

BMI; body mass index, CAR; C-reactive protein-to-albumin ratio, NLR; neutrophil–lymphocyte ratio, PNI; prognostic nutritional index, PMI; psoas muscle index
