# Supplementary material for: Efficacy and survival of nivolumab treatment for recurrent/unresectable esophageal squamous-cell carcinoma: real-world clinical data from a large multi-institutional cohort
Source: Esophagus. 2024 May 8;21(3):319–27. doi: 10.1007/s10388-024-01056-w (PMC11199269; doi:10.1007/s10388-024-01056-w)
Supplement: Supplementary file 3 — Supplementary file3 (DOCX 15 kb) [file 10388_2024_1056_MOESM3_ESM.docx]

eTable 3. Details of Treatment-related Adverse Events

| No. (%) | n=282 | | | | |
| --- | --- | --- | --- | --- | --- |
|  | Grade 2-5 | Grade 2 | Grade 3 | Grade 4 | Grade 5 |
| Treatment-related adverse events  　All events  　Immune-related adverse events  　Rash  　Hypothyroidism  　Interstitial lung disease  　Lung infection  　Diarrhea  Abnormal hepatic function  　Hyponatremia  　Fatigue  　Glucose intolerance  　Abnormal renal function  　Peripheral neuropathy  　Decreased appetite  　Adrenal failure  　Sialadenitis of the submandibular gland  　Bile duct infection  　Hypertension  　Mucosal lesions of the oral cavity  　Cardiac insufficiency  　Cholestatic jaundice | 74 (26.2)  51 (18.1)  12 (4.3)  12 (4.3)  10 (3.5)  9 (3.2)  4 (1.4)  4 (1.4)  3 (1.1)  2 (0.7)  2 (0.7)  2 (0.7)  2 (0.7)  2 (0.7)  1 (0.4)  1 (0.4)  1 (0.4)  1 (0.4)  1 (0.4)  1 (0.4)  1 (0.4) | 48 (17.0)  37 (13.1)  12 (4.3)  11 (3.9)  3 (1.1)  5 (1.8)  3 (1.1)  3 (1.1)  0  1 (0.4)  2 (0.7)  1 (0.4)  1 (0.4)  2 (0.7)  0  1 (0.4)  1 (0.4)  0  0  0  0 | 24 (8.5)  12 (4.3)  0  1 (0.4)  7 (2.5)  4 (1.4)  1 (0.4)  0  2 (0.7)  1 (0.4)  0  1 (0.4)  1 (0.4)  0  1 (0.4)  0  0  1 (0.4)  1 (0.4)  1 (0.4)  1 (0.4) | 2 (0.7)  2 (0.7)  0  0  0  0  0  1 (0.4)  1 (0.4)  0  0  0  0  0  0  0  0  0  0  0  0 | 0  0  0  0  0  0  0  0  0  0  0  0  0  0  0  0  0  0  0  0  0 |
